# Plant-Based Meat Analogues from Alternative Protein: A Systematic Literature Review

**DOI:** 10.3390/foods11182870

**Published:** 2022-09-16

**Authors:** Izalin Zahari, Karolina Östbring, Jeanette K. Purhagen, Marilyn Rayner

**Affiliations:** 1 Department of Food Technology Engineering and Nutrition, Lund University, Naturvetarvägen 12, 22362 Lund, Sweden; 2 Malaysian Agricultural Research and Development Institute (MARDI), Serdang 43400, Selangor, Malaysia; 3 Science and Innovation Center, Oatly AB, Ideon Science Park, Scheelevägen 19, 22363 Lund, Sweden

**Keywords:** systematic literature review (SLR), bibliometric analysis, meat analogues, meat substitutes, high-moisture meat analogues (HMMA), texturized vegetable protein (TVP), plant-based protein, alternative proteins, texturizing technique, extrusion cooking

## Abstract

This study aimed to conduct a systematic literature review (SLR) of the research performed in the plant-based meat analogues area. Historical, current, and future tendencies are discussed. The paper offers a comprehensive SLR coupled with a bibliometric analysis of the publication from 1972 to January 2022. The articles were obtained using a research string and precise inclusion and exclusion criteria from two prominent databases, Scopus and Web of Science (WoS). The Preferred Reporting Items for Systematic Reviews and Meta-Analyses (PRISMA) flow technique was used to describe the data screening and selection. In total, 84 publications were selected for further analysis after a thorough literature assessment. From this study, six main themes were identified: (1) objectives of the study; (2) type of plant protein; (3) product type; (4) added ingredients; (5) texturization technique; and (6) quality assessment considered in the studies. Recent trends in publication imply that meat analogue technology is gaining prominence. This review revealed significant research on improving meat analogues via texturization. Even though extrusion is used industrially, the technique is still in its infancy and needs improvement. Future studies should focus more on fiber and protein–protein interactions, macromolecule conformation and mechanisms, diversifying or improving current methods, sensory attributes, and gastrointestinal absorption rate of each novel protein ingredient.

## 1. Introduction

Meat is a major source of dietary protein. It is frequently recognized as a high-quality protein source due to its nutritional qualities and favorable sensory properties such as texture and flavor. However, a rising global population has led to a rise in the production and consumption of meat around the world [1], which has raised environmental concerns regarding the usage of land and water, as well as the impact of pollution and climate change, greenhouse gas emissions, and the loss of biodiversity [2]. The inefficient resource usage per gram of protein consumed and greenhouse gas emissions from animal farming have led to recommendations to minimize meat consumption. As the number of European vegetarians, vegans, and flexitarians limiting their meat consumption has increased over the few last years [3,4,5], the market for plant-based meat is expected to reach a key milestone of $30.9 billion by 2026 [6]. In addition, religious concerns and expensive production costs [7,8] also contributes to the transition from animal protein to non-animal protein diets. Plant proteins seem to be a possible solution to these issues since they can replace meat through the creation of nutritionally and structurally equivalent meat-like products. These products are referred to as meat substitutes. Some terminology for meat substitutes includes meat replacers, meat analogues, meat imitations, nonmeat protein alternatives, meatless meats, man-made meats, artificial meats, meat-like meats, mock meats, faux meats, and fake meats. These can be partial or full substitutes for meat, and there is an extensive range of textures. The term “meat analogue” usually refers to products that have a similar look, texture, taste, and color to meat but do not include any meat [9,10].

Over the years, the application of several protein texturizing techniques has been extensively investigated in order to produce meat analogues or meat substitutes. The concept was first developed in the 1970s in an attempt to develop a texturizing technique by using several types of proteins, mainly soy. Isolated soy protein was added to defatted brewers’ yeast to boost protein content and improve the texture of meatball, wiener, and hamburger formulations [9]. This idea has led to the development of several research areas. Among them are studies conducted by Taranto [11], who evaluated the function of the extrusion screw in texturized soy and glandless cottonseed flour, and Byun [12], who showed that soy protein isolates admixtures can be spun using lab-designed equipment. This initial endeavor resulted in several types of resembling meat, so-called meat analogues (moisture content of 50–80%) and texturized vegetable protein (moisture content below 30%). TVP is often combined with real meat to stimulate the texture and appearance of real meat. While HMMA has a fibrous structure, it may be used as a whole meat substitute, which is gaining popularity in the food industry [13].

**Figure 1:**
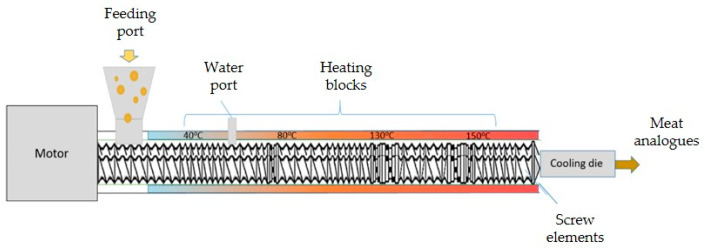
Illustration of high-moisture extrusion (HME) with twin-screw extruder adapted from Zahari et al. [14].

**Figure 2:**
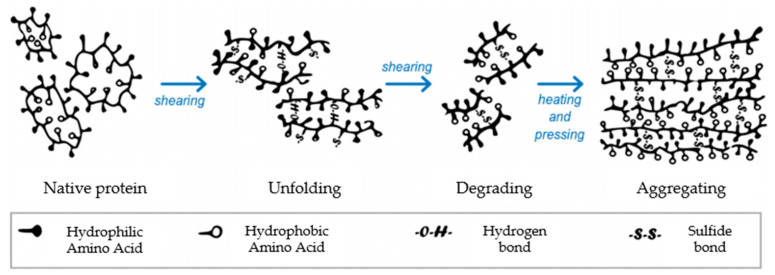
Changes in protein conformation during extrusion of HMMA. Modified illustration adapted from Zhang et al. [15].

**Figure 3:**
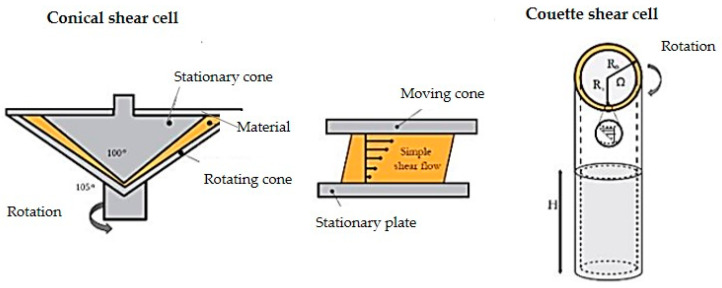
Illustration of conical shear and Couette shear cell adapted from Dekkers [16].

**Figure 4:**
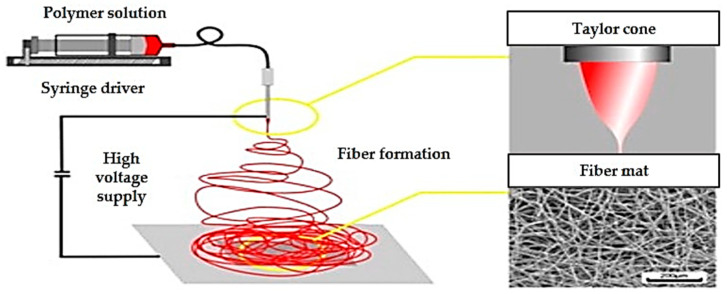
Illustration of electrospinning process adapted from Nieuwland et al. [18].

In manufacturing HMMA utilizing extrusion technology, protein is the most crucial element. Because of the mix, shear, heat, pressure, and cooling (Figure 1) obtained in the extruder and cooling die, the protein is denatured, unfolded, realigned, and cross-linked during extrusion (Figure 2) [14]. Protein interactions are regulated by additional factors, such as protein type, pre-treatment, and extrusion parameters, in addition to the types of protein bonds present in HMMA [15]. Past studies have shown that shearing a protein dispersion in a cone-shaped shear cell may create finely fibrous structures that mimic flesh. Couette cells (Figure 3) with a rotating inner cylinder replaced shear cells (batch systems) for semi-continuous operations [16]. High-temperature shearing of protein mixtures in a wide-gap Couette shear cell has also led to anisotropic, meat-like structures. Rotation causes a simple shear flow in the protein dispersion, which aligns the proteins and causes solidification. Compared to extrusion cooking which is a continuous process and is faster, the shear cell technique in a batch process may create large, thick, fibrous particles but requires at least 20 min of residence time, while the Couette cell can be operated in a continuous mode and is easy to handle [17]. On the other hand, the spinning technique is a complicated process that spins high-concentration plant protein into thin fibers that mimic meat (Figure 4). The high voltage between the nozzle and the grounded collector drives electrospinning. In addition, this delicate and costly method uses acid/alkaline solvents and water, causing considerable waste. Electro-spinning mixes protein with other polymers based on solubility, viscosity, conductivity, and other factors [18].

In terms of plant protein sources, soybeans were widely employed in the beginning (the 1970s) as a meat substitute such as tofu and tempeh, while wheat gluten was renowned for seitan [19]. It was reported that in 1976, a total of about 38,700 metric tons (MT) of texturized soy protein (TSP) was produced, which was equivalent to 14% of the total amount of soy flour produced for human consumption [20]. According to Taranto et al. [11], McAnelly (1964) textured defatted soybean flour by pre-moistening the flour before exposing it to steam and pressure heating. Texturized protein products were then created by Taranto using extrusion and non-extrusion techniques from defatted soy and glandless cottonseed flours. To this day, researchers have focused their efforts on generating the most palatable meat substitutes from alternative protein sources other than soybeans. These meat substitutes may be created using alternative protein on their own, or with soy protein or wheat gluten added to the mix. Additionally, there has been an increase in the number of studies examining not only the various types of protein materials and texturizing techniques, but also the internal mechanisms, chemical and physical properties, nutritional values, protein interactions, and fiber formation that underpin the mechanism of the products produced [21,22,23,24,25,26,27].

Various review articles have been published on meat analogues and TVP processing from different angles. In terms of extrusion, Zhang and colleagues [28] reviewed 115 articles on TVP production, which focused on the effects of barrel temperature, moisture content, feed rate, and screw speed on TVP quality. Other extensive discussions on extrusions have been done by several authors, which covered the aspects related to raw materials, functional properties, and physicochemical changes during the process [29,30,31,32]. Several techniques used to make fibrous products that mimic muscle meats were discussed by Dekkers et al. [33]. Furthermore, there has been a long debate about the molecular, nutritional, and functional properties of alternative protein sources as compared to meat [34]. Some reviews focused on the ingredients used in meat analogues including plant by-products [35,36] and also compared them with traditional meat products [37,38]. An interesting study that reviewed advances in the physical functionality of proteins from non-animal sources in the past three years concerning their potential as meat analogues was carried out by Owens and Levary [38]. The reviews in recent times have focused on more specific aspects of meat analogues, such as consumer acceptance [39], structure design [10], additive ingredients applied in meat analogues [40], and methods for testing the quality of meat analogues [41,42].

This study aimed to conduct a systematic literature review (SLR) of the research conducted in the plant-based meat analogues area. The purpose of this review was to provide a comprehensive overview of the rapid growth of emerging protein texturization technologies, which need periodic evaluations to keep researchers up-to-date. Our contribution of this paper to the scientific literature is a thorough, up-to-date assessment of the evolution of a large variety of plant-based proteins. The comprehensive literature review was analyzed using bibliometric networks to investigate the relationship between authors and content. Thus, a literature review on meat analogues was conducted to answer the following research questions:

**RQ1.**  To what extent has research been conducted on the development of meat analogues derived from plant protein-based materials?
**RQ2.**  What are the key research themes in the literature on plant-based meat analogues?

This review may assist in linking information across several main elements and also in introducing new ideas and encouraging innovation. This paper is divided into the following five sections: An introduction is provided in Section 1, and the research methodology is described in Section 2. The findings and outcomes for the first question (RQ1) investigated in this article are presented in Section 3.1, Section 3.2 and Section 3.3. The second research question (RQ2) is addressed in Section 3.4. Some recommendations for future studies are suggested in Section 4, and the conclusion is provided in Section 5.

## 2. Methodology

### 2.1. Overview of Review Protocol

**Figure 5:**
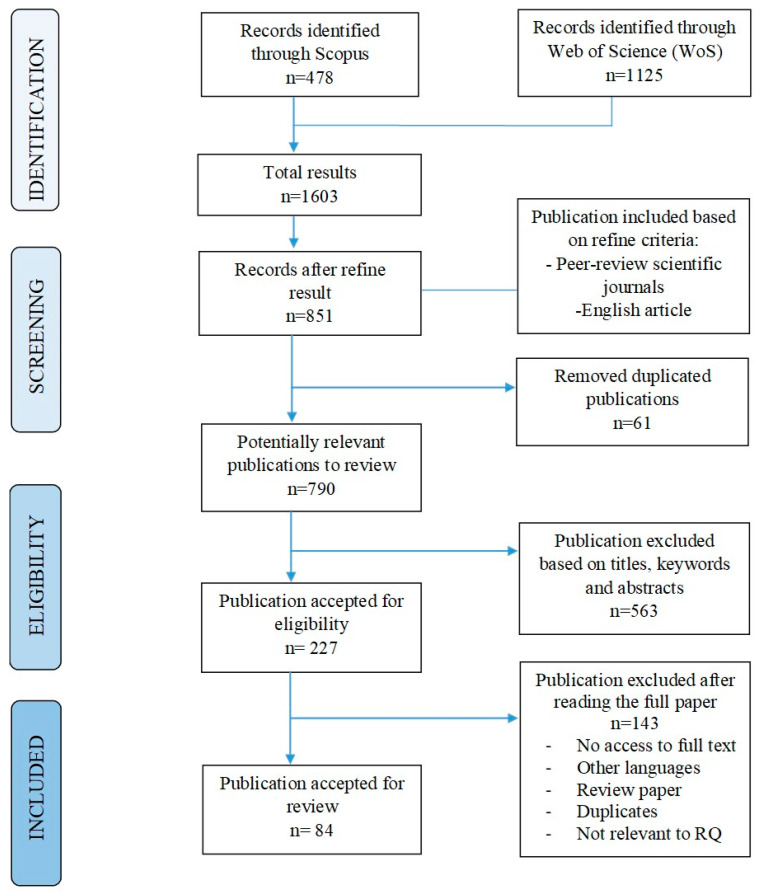
Flowchart of the systematic review search process based on PRISMA guidelines set out in Moher et al. [45].

A systematic literature review (SLR) is one way to perform a more complete study of the present body of information. SLR aims to discover and synthesize all relevant research in an organized, open, and reproducible manner at each stage of the process. Its ultimate goal is to conduct a full search and analysis of the relevant research [43]. A comprehensive approach known as PRISMA (Preferred Reporting Items for Systematic Reviews and Meta-Analyses) was used to conduct a systematic literature review that specifies the process of study selection and rejection (Figure 5). This method has been effectively used in a variety of academic fields and can rapidly synthesize key discoveries from an existing knowledge base [44].

### 2.2. Literature Retrieval and Selection

To answer the research questions (RQs), the advanced retrieval functions in the Scopus and Web of Science (FSTA resources) collection databases were used to retrieve the relevant papers according to the criteria. A search string (Table 1) was developed to ensure the quality of the literature. This study was performed in 2019 and was periodically updated until January 2022. The preliminary search yielded 1603 records. For inclusion criteria, the document categories were firstly limited to peer-reviewed scientific journals and English-language articles, excluding forms such as conference papers, proceedings, book chapters, reports, and editorial materials. A total of 851 records was left after this first-round inspection. Next, data from accepted articles were loaded into Endnote (online bibliographic management software), version basic, Clarivate Analytics (https://access.clarivate.com/#/login?app=endnote, accessed on 1 February 2022). Endnote software facilitates duplicate removal. After removing the duplicates (61 articles), the remaining articles was retrieved and evaluated for relevance by carefully reading the title, keywords, and abstract.

The review’s scope included 227 articles, and the content was evaluated by reading the full article (abstract, method, results, and conclusion). Following that, 143 articles were eliminated as undoubtedly out of the review’s scope. Review articles and those without the full text were removed. At this stage, there were still several articles written in languages other than English (the titles were the only part that was translated), and duplicates that needed to be eliminated. The inclusion and exclusion criteria for this round focused on whether the document was consistent or not with the research questions, which were: (i) studies focused on textured vegetable protein (TVP) or meat analogues processing from plant-based protein (non-animal based which include fungi, algae, and mushroom); (ii) studies focused on quality assessment of protein materials or product developed or both. In particular, the following topics were excluded: (i) non-TVP or non-meat analogues products such as expanded products (snacks, cereals, and pasta); (ii) studies involving animal-based products (meat, milk, fish, blood, whey) or insects based raw materials added in TVP or meat analogues; (iii) other textured products such as 3D printed products, cell-cultures products, food ingredients, flour, cookies, bakeries, pet food, drugs, and non-food products; and (iv) unrelated subjects such as studies mainly on consumer acceptance, sensorial techniques, economic aspects, or marketing (without the texturization process). To be clearer, studies were only selected based on plant protein materials, processing into texturized protein, and/or meat analogues. There were also some articles that were discovered to be totally unrelated only after reading the full articles. In total, 767 records were removed, leaving 84 full-length articles in the review portfolio.

### 2.3. Bibliometric Analysis

To investigate the chosen relevant publications, bibliometric techniques such as co-authorship analysis and keyword co-occurrence analysis were used to trace the knowledge architecture of a particular study subject. It is also a technique for detecting the patterns in development or the directions in which future research should go [46]. These are the most thorough procedures utilized to illustrate the relationship between the author and the keyword cluster used in the selected articles. VOS viewer software was used to perform the clustering. The VOS acronym stands for “visualisation of similarities”, and the program is an open-source application (www.vosviewer.com accessed on 1 February 2022) developed to create and visualize bibliometric maps [47]. It has been shown that network visualization is a valuable tool for analyzing a wide range of bibliometric networks, such as networks of co-authorship relationships among academics, and networks of keyword co-occurrence associations in published works [48].

## 3. Results and Discussion

### 3.1. Publication Trends

**Figure 6:**
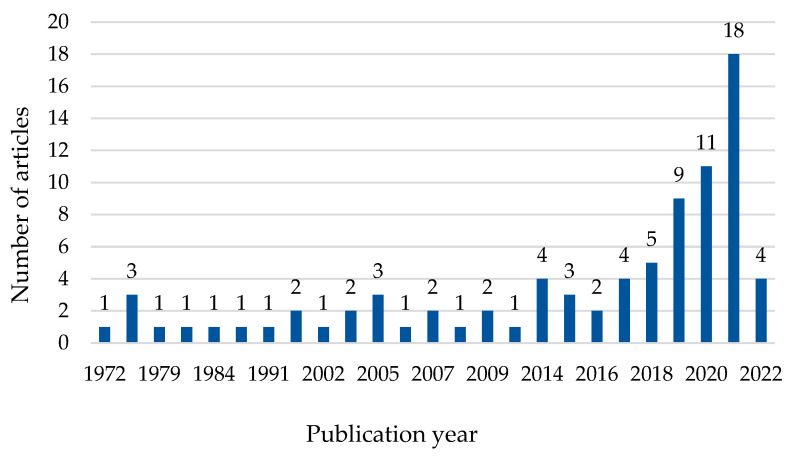
Annual publication trend of 84 papers between 1972 and January 2022 retrieved from Scopus and Web of Science (WoS).

Figure 6 illustrates the number of articles based on the publication year, which varied across the years. The first publication on the texturization of plant protein for meat substitutes appeared in 1972, and the findings revealed that there has been a substantial quantity of scientific production since then. It particularly peaked in 2021 when 18 papers on the topic were published, a trend that may continue in the coming years ahead, considering that the database search for 2022 only included papers available until January.

### 3.2. Journal-Based Publications

Table 2 presents the list of the most prominent journals (top 13) publishing articles on meat analogues or TVP and development in the field. The bibliometric analysis revealed that the 84 papers included in the systematic review were published in 38 journals between 1972 and 2022. It was revealed that the most frequent journals chosen by the authors were *Foods* with 15 articles [14,15,24,41,49,50,51,52,53,54,55,56,57,58,59], followed by *Journal of Food Science* with nine articles [11,20,25,60,61,62,63,64,65]. Because the journals were indexed by both Scopus and Web of Science databases, they were all of high quality, and most were placed in Quartile 1 or 2 (see Table 2).

### 3.3. Meat Analogue Bibliometric Networks 

#### 3.3.1. Network Visualization for Author Keywords

**Figure 7:**
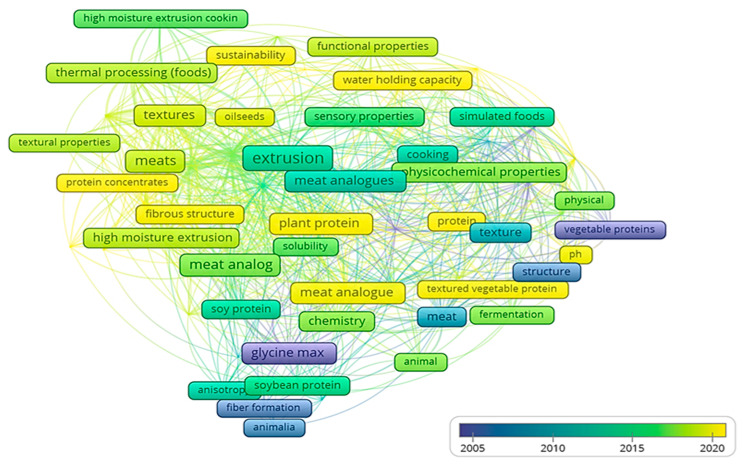
Overlay visualization based on keyword re-occurrence of at least three times based on 84 retrieved articles.

In the network presented in Figure 7, each circle represents a keyword. A node depicts a term in a publication title or abstract. The size of a node indicates the number of publications in which it is present. The nodes are colored based on this journal’s average year of occurrence using the color scheme depicted in the legend. Nodes closer to each other are more similar than nodes further apart. The thickness of a link between two nodes indicates the likelihood that they co-occur in the same publication. The minimum number of occurrences of the keywords is three. From 594 keywords, 71 meet the threshold. It was found that a number of authors utilized variants of keywords. The most frequently occurring terms were “extrusion” (27 documents), “protein” (25 documents), and “meat” (16 documents), followed by the terms “moisture” (15 documents), “meat analog” (14 documents), “plant protein” (13 documents), “meat analogues” (11 documents), and “meat analogue” (10 documents). Early in the twenty-first century, there was an increased interest in “soy proteins”, “glycine max (soy bean)”, and “triticum aestivum (wheat)”, as well as “flour”, “vegetable protein”, “gluten”, and “plant protein” (average publication year: 2005–2010). In recent years, authors have increasingly focused on “extrusion”, “meat analogues”, “high moisture extrusion cooking”, “functional properties”, and “mechanical properties” (average publication year: 2015–2020).

#### 3.3.2. Network Visualization by Author

**Figure 8:**
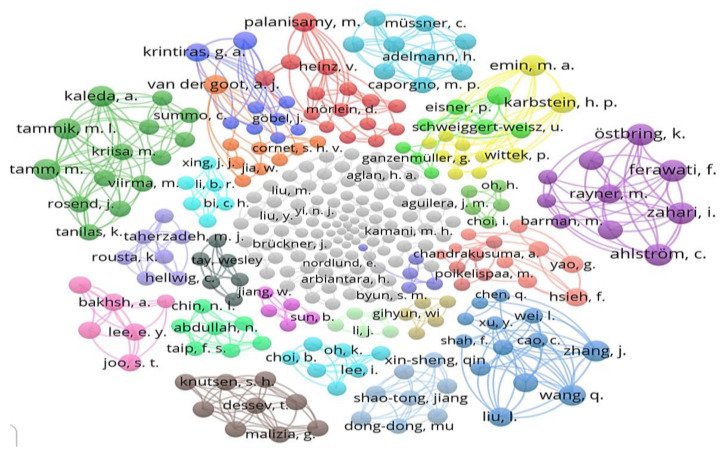
Network visualization based on authors in 84 retrieved articles.

Our search revealed that 297 authors contributed to the total number of publications based on the selected 84 articles. Figure 8 depicts a network visualization of the authors’ co-occurrence network for all the publications analyzed in this search, which formed 56 clusters. Each circle indicates a different author’s name. The size of the circle is proportional to the number of articles each author has written on this subject. In general, the greater the proximity of authors in the visualization, the stronger their bibliographic connection. Only 43 authors had two or more publications, whereas 254 authors had only one publication. Emin, M.A.; Hsieh, F.; and Kabstein, H.P. had the most publications in this dataset with five each, followed by Yao, G. with four publications. In addition, the co-authorship network’s largest connected subgraph included just 18 authors, including Van der Goot, A.J.; Krintiras, G.A.; Cornet, S.H.V.; and Jia, W. The second important subgraph in the co-authorship network consisted of 15 authors, including Grahl, S.; Palanisamy, M.; Saerens, W.; and their peers.

### 3.4. Classifications

One of the most important things to report on in SLRs is the table of findings. The table clearly explains the results and makes them easy to understand [44]. Table 3 shows all selected articles, which were classified according to the content, resulting in six main themes: (1) objectives of the study; (2) type of plant protein; (3) product type; (4) added ingredient; (5) texturization technique used; and (6) quality assessment considered in the studies. These six themes further produced 22 sub-themes, which provided answers to the second research question (RQ2) of this SLR. The background of the selected studies is discussed in the following section.

#### 3.4.1. Objectives of the Reviewed Studies

**Figure 9:**
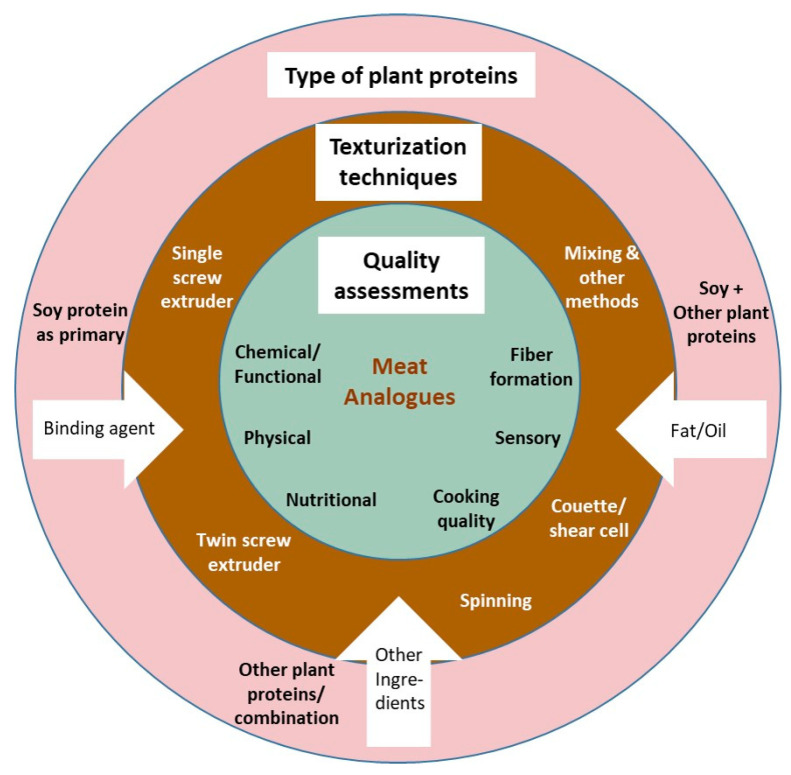
Overall framework composed of three components.

The articles chosen were well-balanced in terms of their objectives. Table 3 demonstrates that the majority of the included articles studied the texturization technique in creating texturized vegetable protein (TVP) or meat analogues (MA), as well as protein materials using either commercial soy protein or other protein-based materials. Some studies investigated how fiber development evolved in the MA/TVP. Another critical aspect of meat substitutes is quality assessment, which was extensively identified in many articles. We proposed a framework to address three main aspects highlighted in the reviewed articles, as seen in Figure 9.

#### 3.4.2. Type of Plant Proteins Used

##### Soy Protein as Primary Component

In this first sub-theme, it was discovered that experts had focused their attention on soy protein (either in the form of flour, concentrate, or isolate) as the primary component in textured protein products for years. Soy protein is used either by mixing it directly in the formulation of meat substitutes together with other ingredients, or by processing it via texturizing techniques into TVP or HMMA. Soy garnered interest for its protein quality and because it has satisfactory functional qualities (such as the ability to absorb water and oil and its emulsifying properties), and it has been used in the production of a variety of unique meat substitutes. As a result of the excellent features that soy has, it is usually used as a standard or benchmark to compare different protein materials [78] and as a model to explore many other aspects of meat analogues [63], texturizing techniques [19], extrusion parameters [15], and product structure [58,59,73]. Dahl and Villota [60] used soy flour altered with acid (HCl) or base (NaOH) and studied the pH effects on the functional properties of soy protein. Liu and Hsieh [88] used two commercial soy protein isolates to study the fibrous meat analogues produced through high moisture extrusion or gels via heating and chilling, with different concentrations and/or temperatures. Due to its lower cost, soy protein concentrates (SPCs) are widely utilized as an alternative to soy protein isolate (SPI). Pietsch et al. [100] reported that SPCs may produce more prominent anisotropic (properties of materials depending on the direction) structures than SPI. Two other studies using high-moisture extrusion of soy meat analogues (SPCs) were conducted by Palanisamy et al. [98] and Chiang et al. [72].

##### Soy Protein Combined with Other Plant Proteins

Researchers started to use commercial soy protein isolates/concentrate together with other protein sources in order to reduce the use of soy protein, and also to study their combination, establish texturization conditions, and to aim to diversify meat products in the market with different formulations, as discovered by many authors [15,61,62,70]. Kozlowska et al. [81] used high- and low-pressure processing to texturize the flours and concentrates that were derived from soybean and rapeseed, as well as the blends of soybean and rapeseed (1:1). When combined with additional plant proteins, the extruded meat analogues were found to be of higher quality. Numerous researchers have found that by combining soy protein and wheat gluten, meat substitutes might match the texture, color, flavor, and function of red meat, as well as enhance the disulphide bonds to generate a fibrous structure [27,72,111]. In the investigation of the total heat transfer coefficient in extrusion processing conducted by Lee et al. [84], meat analogues were mixed with another established protein and wheat starch. In a separate investigation, Liu and Hsieh [89] and Ranasinghesagara et al. [25] similarly co-extruded soy protein with wheat gluten and starch to produce fibrous meat analogues under high-moisture and high-temperature conditions. According to prior studies by Neumann et al. [95], non-heated corn gluten (CG) demonstrated superior functional performance compared to heat-dried corn gluten meal. Thus, wet-milled corn gluten and defatted soy flour (DSF) were combined and extruded to produce textured meals afterwards. Hemp protein could also be mixed with soy protein isolate up to 60% in the formulation of high moisture meat analogues, as reported in a previous study [15]. In the context of restructured meat analogues, mushrooms, which contain high levels of sulphur-containing amino acids, and glutamic acid, which implies a distinctive umami taste, are the materials that are mostly used, as they closely resemble those with a natural meaty flavor and texture. Because of these similarities, mushrooms have been employed by mixing directly with other added ingredients in the formulation of several types of restructured meat analogues [66,77,80,94]. Other plant proteins, which were recently found to be promising to partially substitute soy protein in meat analogues, include spirulina [76], yam [87], rice protein isolate [85], and microalgae powder [71].

##### Alternative Proteins without Soy Protein

The second sub-theme pertained to alternative plant proteins used in previous studies. Researchers examined other protein resources to completely replace soy protein in the formulations. This interest is due to other factors, including GMO issues, allergies, and an unfavorable climate for soy cultivation. However, in thermomechanical processing that involves texturizing equipment, it is impossible to make great meat analogues without the use of components that have a high percentage of protein. To develop a comprehensive fibrous structure similar to actual tissue, extrusion, shear cell, and spinning technologies, for example, need ingredients with a high protein concentration. Several studies showed promising protein ingredients such as pea protein [31,93], mucuna beans [96], peanut protein [103,114], and faba beans [24,105]. The majority of the plant-based proteins (such as those found in legumes and oilseeds, for example) contain undesirable components such as anti-nutrients (glucosinolates, phenolic compounds, and phytic acid) and inhibitors of digestive enzymes. These components reduce the nutritional value and acceptability of plant-based proteins and impart an unpleasant flavor such as bitterness. To be accepted by consumers as meat analogues, these undesirable components must be eliminated by certain pre-treatments before being used. Those treatments used together with the protein extraction process, on the other hand, lead to a loss of functional qualities as well as reduction of the protein’s quality and quantity. This is by far the most challenging obstacle to overcome when researching and developing novel plant protein materials. Because various plants have varying protein types and qualities, numerous efforts have been made to create a novel blend of several plant proteins with the hope that some proteins may compensate for the drawbacks of other plant proteins during the texturization process. For example, Kozlowska et al. [81] and Zahari et al. [14] suggested that rapeseed protein would be a good source for supplementing other vegetable proteins, e.g., soybean and yellow pea. Arueya et al. [67] created meat analogues from Lima bean protein concentrate (LBPC) and African oil bean seed concentrate (AOBSPC), which are underutilized legumes with high nutritional potential grown mainly in Peru. Similarly, De Angelis et al. [49] successfully developed meat analogues by employing different protein mixtures from dry fractionated pea and oat protein. In summary, meat analogues produced with other plant proteins than soy have a distinct fibrous structure, high levels of vital amino acids, and a nutritionally useful composition, making them prospective future elements.

#### 3.4.3. Product Type

Two sub-themes were developed under this theme: texturized vegetable protein (TVP), and meat analogues (MA). Regarding nomenclature, there is a considerable degree of disagreement among professionals, with some arguing that extrudates from extruders cannot be referred to be meat analogues, while others disagree. Some authors suggest that the extrudates are not intended for immediate consumption but rather as meat extenders that will be sliced and combined with other substances to form a restructured meat substitute. Thus, depending on the publication, some of the studied articles referred to the extruded product as extrudates or meat analogues, while others referred to it as TVP and utilized it as a replacement for meat. We categorized the terms based on the information provided in the articles, including the final restructured meat-substitute products, which are normally referred to as nuggets, sausages, or patties and may contain added ingredients according to the formulations.

##### Texturized Vegetable Protein (TVP)

In 1978, Hashizume [19] studied a traditional method of manufacturing Koritofu to convert protein into a textured product using a freezing method. In comparison to the other temperatures that were evaluated in the research, such as −5 and −70 °C, it was claimed that the temperature of −20 °C was the one that successfully created the spongy protein that could be utilized as a replacement for animal flesh. According to Kozlowska et al. [81], who used two different models of extruder, the high-pressure technique produces a product with a specialized purpose as a meat extender, whereas the low-pressure technique produces a product that is suitable for developing meat analogues. However, Neumann et al. [95] defined the product as TVP when it was produced by low-pressure extrusion at pressures around 100–200 psi. On the other hand, according to Maung et al. [92], TVP is often used as a meat extender or directly as meat analogues in hamburger patties, sausages, steak, sliced meats, and many other products. Bakhsh et al. [41] recently revealed that TVP, when used as a major ingredient in hamburgers, had characteristics comparable to those of hamburgers made from beef and pork. However, it was noted in his other study that the surface of the patties that were made from TVP and texturized SPI both had a granular look, which is a downside of employing those two ingredients [68]. While using chickpea flour and TVP, Sharima-Abdullah et al. [107] produced an imitation of chicken nuggets, which was stated to be a promising product.

##### Meat Analogues

In terms of meat analogues, several researchers developed the products directly as whole muscle meat (mostly from extrusion) with or without some added ingredients [24,49,51,52], but others referred to meat analogues as restructured meat products such as Turkish dry fermented sausages (“sucuk”) from wheat bulgur [69], SPI sausage [79], and edible mushroom sausages [57,109]. The study by Rousta et al. [56], who investigated the culture of the fungus on oat flour and its use in the development of burger patties, demonstrated the productive potential of the fungus for the manufacture of nutrient-dense foods. Saldanha do Carmo and colleagues [105] used response surface methodology (RSM) to optimize the manufacturing of meat analogues made entirely of faba bean protein concentrate acquired by a dry-fractionation technique, which also showed promising results.

#### 3.4.4. Added Ingredient Used to Improve Texturized Products

##### Binding Agents

When making textured vegetable protein products or meat substitutes, it is a common practice to use some amount of additives or chemicals in order to expand the range of raw materials suitable for use in production [84]. Many different binding agents have been utilized as fat replacers to increase the quality of TVP or restructured meat (for example, sausage, nugget, and patty). This has been done to improve the taste, juiciness, mouthfeel, and other sensory qualities. Examples of such additions are starch, fibers, soy and milk proteins, a variety of hydrocolloids, and egg solids. Because the scope of this study is limited to plant-based products, we will not include any materials derived from animal sources; as a result, we will only count a few studies. Arora et al. [66] investigated the effect of various quantities of binding agents (carrageenan, soy protein concentrate, casein, and xanthan gum) on the qualitative features and nutritional qualities of mushroom-based sausage analogues prepared with 5% saturated fat. Carrageenan (0.8%) had the greatest outcomes in terms of minimizing purge loss, cook loss, and emulsion stability, all of which improved the process output. It was reported that methylcellulose (MC) is an effective binder, particularly for the meat analogues that do not need to be preheated for gel formation, because of its one-of-a-kind thermal gelling ability and emulsifying qualities [41]. Contradictorily, Sakai et al. [26] developed an alternative new binding mechanism, since chemicals are used in the production of methylcellulose. The results suggest that the protein–sugar beet pectin crosslink catalyzed by laccase may serve as a binding mechanism for the TVP patties. In addition, microbial transglutaminase (TG) and sodium alginate (AL) are two binding agents that are often employed in food preparation. Each of these binding agents functions in distinct ways for protein binding or gelling systems [26,69,86]. The authors suggest that the combination of TG and AL may synergistically affect the eating quality of soy patties, although more improvements are required. While AL has an advantage in the creation of restructured meat because it can create a thermostable and irreversible gel (in the presence of Ca2+), TG has been used as a cold-set binder since it catalyzes covalent bonds between the ε-amino group (a primary amine) of peptide-bound lysine and the γ-carboxamide group of peptide-bound glutamine [26,69].

##### Fat/Oil

In order to reduce the saturated fatty acid and cholesterol levels of certain restructured meat substitutes, animal fats were substituted by vegetable oils such as olive oil [26,69], palm oil [55], canola oil, and coconut oil [41,68]. Depending on the raw materials, oil is used in different amounts to obtain a more meat-like texture and to increase the flavor, juicy quality, tenderness, and several other qualities of MA related to sensorial experience. For example, Mazlan et al. [55] used 10% palm oil in the soy–mushroom extrusion mixture. Kamani et al. [79] used 8% oil in SPI-gluten sausage analogues, Bakhsh et al. [68] used a total of 7.5% of oil in the TVP patty formulation, while Saerens et al. [104] used fat emulsion from pea protein and rapeseed oil in the soy and pumpkin seed protein-based burger patty formulation. It has been observed that fat has an influence on thermal–mechanical processing as a lubricating agent and helps to accelerate the creation of protein alignment networks. Recent research conducted by Kendler and colleagues [53] investigated the impact that oil (0–6%) had on the extrusion-relevant parameters and structure-related properties of extruded wheat gluten. According to the results, using oil in the high-moisture extrusion led to a significant change in the process conditions, as well as in the rheological properties and product qualities. The oil concentration and addition point were discovered to have an impact on the size of the oil droplets. The size of the oil droplets became larger as the oil content increased, indicating that the fat droplets were subjected to coalescence. On the other hand, there was a difference in oil droplet size depending on where in the extruder the oil was injected, where injection at the end of the extruder resulted in smaller oil droplets [53]. The anticipation that oil droplet breakage is improved at greater matrix viscosities was supported by these findings. Some protein materials still contain a high content of oil, such as rapeseed protein concentrate [14], which could thus enhance the final product characteristics without adding any fat during thermal–mechanical processing. Nevertheless, too much oil may contribute to the lubricating effect (slippery condition) within the barrel, hindering the protein denaturation process.

##### Other Ingredients

In addition to binders and lipids, there are a few other ingredients that are normally added into the formulation of meat analogues, especially restructured meat. For extruded meat analogues, added ingredients such as polysaccharides, colorants, flavoring, and seasoning were used during the cooking process. Exogenous polysaccharides are one of the key additions often employed in the food industry to increase the functional qualities of food proteins and optimize texture, and they were utilized to study the impact of polysaccharides in the meat analogues of peanut protein [115]. Since the majority of extruded meat analogues lack flavor and color, adding additional ingredients such as meat flavor powder and red yeast rice is required when incorporating them into restructured meat, as shown in [57]. Wen et al. [110] studied the effect of calcium stearyl lactylate (CSL) in extrusion processing and found that CSL has the potential to greatly improve the extrudates’ textural qualities, including the number of fibrils present and the size of their pores. In terms of restructured meat, the taste of patties and sausages was improved by the addition of colorant, sugar and salt, flavoring, seasoning, herbs, and spices such as cumin, cinnamon, pepper, and garlic [57,68,69,109]. According to Yuliarti et al. [113], the inclusion of calcium chloride and baking powder in the formulation of pea and wheat protein nugget was to boost the protein’s ability to bind water and to create air cells in the dough, which might improve the fibrous structure. Carotene and anthocyanin are also being added to enhance the vegetarian sausage analogues; nevertheless, it has been noted that their levels drop with storage, thus requiring additional development [109].

#### 3.4.5. Type of Texturization Technique

##### Extrusion

Many texturization technique studies have been conducted in recent years, which were classified into five groups: single screw extrusion (SSE), twin screw extrusion (TSE), shear/Couette cell (SC), spinning (S), and mixing/other methods (M/O). For thermal–mechanical processing, high moisture extrusion technology has become a popular method compared to other texturizing methods due to its lower energy consumption, lack of waste to be disposed of, high efficiency, and higher textured product quality [14,21,24,49,51,53,54,59,101,112]. In the past, the production of TVP, which often has a lower moisture content, was mostly done using single-screw extruders [81,96,99,103]. A drawback with TVP is that it must first undergo a rehydration process before being included in the meat substitutes’ formulas. Extrusion seems to be becoming increasingly popular since it can be utilized for both low- and high-moisture products. Because of these factors, several experiments were performed to gain an understanding of the relationship between the processing parameters of the extruder and the final product. According to Samard et al. [106], who examined the effect of extrusion type (low- and high-moisture extrusion cooking) on the physicochemical properties of meat substitutes, the cooling die region of HMMAs is thought to be crucial for the cross-link formation. As we know, for most plant proteins, a certain pre-treatment (acid or alkaline) is typically required during the extraction process in order to receive a higher yield as well as more desirable extrusion outcomes. Furthermore, extrusion was shown to partly break down phytates in a matrix-dependent way, improving the material’s nutritional quality [52]. In addition to the composition of raw materials, various extrusion parameters (screw configuration, temperature set-up, screw speed, solid and liquid dosing, and moisture content), as well as diverse manufacturers, can result in dramatically different product structures and textures, even when using the same raw material. Each processing parameter will influence the product specification during extrusion processing. For instance, most studies investigated the effect of screw speed, water feed, extrusion temperature, and feed rate on the product characteristics [14,78,100,102,106]. Studies showed that water feed was the most influential factor in the extruder process and product qualities, followed by screw speed and barrel temperature [14,97]. Several blocks of barrels could be used in the high-moisture extruder, with the highest temperature ranging from 100 to 180 °C [97,100,102,103,106]. Earlier, Lee et al. [84] calculated the total heat transfer coefficient in a long slit cooling die and discovered that the projected product temperature at the die output was 6.8 °C of the observed experimental value, whereas several authors investigated the effects of the specific mechanical energy (SME; kJ/kg) [14,74,100] and specific thermal energy (STE; kJ/kg) [71] on the physicochemical properties of texturized meat analogues. Increasing the screw speed required more energy, but the SME dropped as the moisture content increased [14,71].

##### Shear/Couette Cell

Several studies have suggested the shear cell as a suitable technique for meat analogues production. A cone–cone device (shear cell) and a concentric cylinder device (Couette cell) were created by Krintiras et al. [17,82,83] based on the notion of a flow-driven structure. In both devices, a model system of soy protein isolate (SPI) and vital wheat gluten mix was employed, resulting in anisotropic structures that may be used as meat replacers. In 2016, they invented a 7 L Couette cell system for making structured soy meat replacer, and high anisotropy fibers were developed. The up-scaled Couette cell can produce 30 mm thick flesh replacers that mimic meat, which means that the research found no impediments to scaling up the idea. The flexible design enables the manufacturing of meat substitute goods in sizes not previously possible, which might be beneficial in replacing chicken breast or beef [17]. According to Jia et al. [78], the formation of fibrous materials in shear cells is favored when plant materials have two different phases that deform and align when sheared. This can be done by mixing purified ingredients with different water holding capacities, such as soy protein isolate and wheat gluten, or they can be found naturally in a single but less purified ingredient, such as soy protein concentrate. They studied the structuring potential of rapeseed protein concentrate (RPC) with and without wheat gluten (WG) for meat analogues synthesis in a shear cell. Both RPC-only and RPC–WG combinations could become fibrous at 140 °C and 150 °C with 40% dry matter; in addition, WG could enhance the fibrous structure and lighten the color [78].

##### Spinning

In 1972, Stanley et al. [108] studied the properties and ultrastructure of rehydrated spun soy fiber and identified structural differences between soy and beef. According to the study, meat contains repeated sarcomeres, connective tissue that affects texture, a sarcolemma or elastic cell membrane, and actin–myosin cross-bridges, whereas spun soy is a uniform, homogenous fiber with disulfide bonds. Byun and colleagues [12] designed bench-scale protein spinning equipment in the laboratory with some modifications to the prior approach in order to determine the viability of spinning mixes of soy protein isolates. The discovered method is based on the unfolding of peptide chains by alkali treatment and the molecular orientation of mechanically spun fiber. Electrospinning is one of the techniques investigated recently by Mattice et al. [91], and they modified the electrospinning parameters to produce zein fibers with uniform width while minimizing ethanol consumption. Even though electrospinning produces tiny individual fibers, the technology used in this research was reported to have a very low throughput and thus faced problems with efficiency.

##### Other Texturization Methods

In addition to all of the particular thermomechanical and texturizing procedures, there were also other methods being used in exploring and developing meat analogues, such as direct mixing [79], freezing [19], planetary roller extruder [98], mechanical elongation, and antisolvent precipitation [91]. According to the findings of this review, the majority of meat analogues that employed mixing techniques with other ingredients was restructured meat products such as nuggets, sausages, and patties [77,79,80,87,107,109]. Furthermore, Nayak et al. [94] developed a meaty-textured soybean by solid-state fermentation using *Rhizopus oligosporus* and dried *Agaricus* mushroom and compared the textural profile of the optimized fermented soybean with poultry meat.

#### 3.4.6. Quality Assessment Considered

As previously shown in Figure 7, improving the qualitative attributes of meat analogues has been the focus of several investigations over the last few decade. These quality efforts are important for developing excellent meat analogues, especially in understanding the formation of the fibrous structure and protein–protein interactions. Meat consumers were less tolerant to plant-based commodities compared to actual meat products a few years ago, and the main reason was the poorer sensory and nutritional value of the plant-based products. Following that, several novel meat substitutes with enhanced flavor and texture from a variety of plant-based sources can be found on the market, the most popular being based on pea and oat. Several elements of meat substitute characteristics, including chemical and functional properties, physical properties, fiber formation, nutritional properties, cooking quality, and sensory evaluation, are being addressed. Here we may observe how meat analogue research contributed to the food industry. Fiber creation has recently been a hot topic in many authors’ research.

##### Chemical/Functional Properties

The most widely used approach in this sub-theme is proximate analysis. All protein powders and meat substitutes are proximately analyzed using international standard methods (AOAC, AACC, ISO), which include moisture, protein, fat, crude fiber, and ash. In extrusion processing, it is necessary to know the moisture content of the protein materials (feed powder) in order to determine the desired moisture content of the final extrudates. Most of the previous work used a conversion factor of 6.25 for soy protein and 5.7 for wheat gluten [31,88,89,114], and the protein was examined using either the Kjeldahl or Dumas combustion methods. In the future, additional conversion factors specific to each crop may need to be employed to obtain more accurate findings. For example, Mariotti et al. [116] proposed a collection of certain conversion factors for various meals, such as 5.5 for soybean, 5.4 for cereals and legumes pulses, and 5.6 for corn and other sources. The study suggested a more exact default conversion factor of 5.6 rather than 6.25, a scientific way to express nitrogen as protein, which is highly relevant when “protein” refers to “amino acids”. Using flame atomic absorption spectrometry, the levels of the microelements such as iron (Fe), zinc (Zn), copper (Cu), and manganese (Mn) in the extrudate samples were analyzed. Many studies investigated protein–protein interactions using the protein solubility approach [32,88,89,114]. Osen et al. [32] studied the establishment of covalent peptide bonding during the extrusion process in order to evaluate the impact that high moisture extrusion cooking had on the protein changes that occurred within the extruder. Moreover, FTIR is commonly used for investigating protein conformation and is capable of accurately measuring the secondary structure of proteins, since each protein may be linked with a unique set of bands and wavenumber intensities [87,110,112,114,115]. SDS–PAGE analysis was commonly used to investigate the degree of crosslinking, thus could determine the molecular weight distribution [26,74,101,111]. Proteins may polymerize into larger aggregates, rendering those proteins too large to penetrate the flowing gel [74]. As explained by Kaleda et al. [52], changes in the content and conformation of proteins significantly impact the capacity of proteins to hold water. Water holding is highly dependent on the presence of polar hydrophilic groups, while the nonpolar side chains of proteins are responsible for determining the oil holding capacity of a material. Oil holding capacity also relies on the physical trapping of oil and may be explained by the material’s microstructure. Because it impacts the quality and production of meat analogues, water, and oil holding capacity are important factors. The greater the holding capacity of a product, the juicier it will be. The water solubility index (WSI) measures the total quantity of a substance that can be extracted using water. Multiple variables, including powder composition and particle size, conformational state of proteins, molecular size, and cross-linking, may impact WSI [52]. Using carrageenan (0.8%) as a binding agent, mushroom sausages exhibited the lowest amount of purge loss (3.56%) after being frozen, which led to a reduction in drip losses caused by the thawing process [66].

Thermal analysis is usually conducted using differential scanning calorimetry (DSC), thermogravimetric (TG), and differential thermal analysis (DTA) to measure the thermal denaturation of protein, as used in several studies [24,24,88,114]. This also helps in setting the correct barrel temperature during the extrusion process and increases knowledge of the raw materials. The barrel temperature must be high enough to allow protein denaturation during the extrusion process. A rheology study may provide some insight into the flow and deformation of protein materials. Many studies reported the rheology results previously [50,60,114]. It can assess the behavior of proteins under shear stress and strain during heating–cooling cycles and act as a predictor of the quality of the finished product (meat analogues) following thermal–mechanical processing [15,24,100]. Emin et al. [73] employed a closed cavity rheometer (with a specified extrusion-like environment) to investigate the critical process parameters that contribute to a substantial change in the response behavior of a plant protein model system, employing vital wheat gluten as a model system. The findings reveal that temperature, water content, shear, and a step change in shear significantly impact the response behavior of proteins.

##### Physical Properties

Physical attributes consist of analyses performed on TVP or meat substitutes. Textural and structural properties using a texture profile analyzer (TPA), cutting strength and tensile strength, and ultrastructural characteristics such as scanning electron microscopy (SEM) and light microscopy analysis are among the most typical tests performed to determine how the texture or morphology are formed inside the product and how they are connected to other properties such as chemical composition, SME, and sensory attributes. Authors mostly used SEM to comprehend the structural properties of meat analogues [17,80,81,95,98]. The important characteristics highlighted using TPA were hardness and chewiness. As a result of cutting through the fibrous meat analogues, it was discovered that the values for the longitudinal cutting strength were significantly higher than the values for the transversal cutting strength [14]. In most cases, the authors either compared the physical characteristics of meat analogues to a reference product of chicken meat and beef or commercial meat analogues. The degree of texturization of SPI-based meat substitutes rises as the SME decreases, according to several studies [72,74]. Kaleda et al. [52] discovered that this is not the case when employing a screw configuration with several kneading and reverse blocks, resulting in greater mechanical treatment. Lower hardness and chewiness in meat analogues were, on the other hand, reported by Fang et al. [74], being correlated with lower SME, contrary to Chiang et al. [72] and Zahari et al. [14]. In the extruder barrel, protein molecules exhibited major structural changes and unfolding, creating ideal circumstances for molecular rearrangement in the subsequent extruder zones. The meat-like fibrous structure was reported to be formed at the cooling die zone junction due to protein phase separation and rearrangement [58].

When determining color, the results are always associated with the color of the raw materials used, and the temperatures at which the food was cooked might influence the product. For instance, meat analogues from soy protein had a lighter color compared to hemp, and the L* values decreased as the proportion of hemp powder in the formulation increased [15]. Some authors agreed that L* and a* values were dependent on extrusion temperature and moisture during extrusion cooking [52,97]. Raising the moisture content would increase the L* values owing to the lower rates of chemical reactions in the protein composite produced with greater water content [24,97]. Other physical properties applied by the authors in this review on TVP and meat analogues are lateral expansion [96], bulk density [49], porosity [67], expansion index [60,70], and rehydration [70].

##### Fiber Formation

Yao and colleagues [65] devised a technique for determining fiber formation in soy protein extrudates with high moisture content. To assess fiber growth, many polarization measurements are required. Because the fluorescence signal is weak, ambient light must be blocked, which is problematic on a production line. Ranasinghesagara and colleagues [63] refined the technology by establishing an image processing method to automatically quantify fiber creation using digital imaging and by inventing a non-destructive imaging approach with real-time quality control [64]. Later in 2009, more sophisticated technology was applied by incorporating it into a rapid laser scanning system, which permits real-time 2D mapping of fiber production and orientation across the sample [25]. Another interesting study was published by Zhang et al. [114], who employed a multiscale approach paired with emerging techniques such as atomic force microscopy-based infrared spectroscopy and X-ray microscopy to make the entire extrusion process visible to illustrate the process of generating a meat-like fibrous structure. Moreover, simulations show that phase separation under temperature or velocity gradients may lead to multilayer structures [93].

##### Nutritional Analysis

In addition to functional and textural qualities, the nutritional composition of meat substitutes is an essential factor to be considered when substituting meat with plant-based analogues. Regarding amino acids, researchers are exploring alternative approaches to meet the FAO’s requirements for meat. Proteins derived from plants are considered nutritionally insufficient because cereals often lack lysine, and legumes typically have low levels of the sulphur-containing essential amino acids methionine and cysteine. As a result, the quality of the nutrients could be increased by mixing two or more protein sources, which enable the mix to meet FAO standards for a particular age group. Several publications [22,24,32,67,70] reported the amino acid composition; however, no publication was found that analyzed the protein quality or protein digestibility-corrected amino acid score (PDCAAS) of the produced meat analogues. This demonstrates that there is currently a lack of information that is readily available. Previously, it was reported that the amino acid composition of soy and sunflower mixed flours may complement each other [70]. According to Osen et al. [32], extrusion did not influence hydrolysis or amino acid composition, possibly because high feed moisture minimized shear stress and mechanical energy loss in the extruder. The main amino acids in the WG/SPI meat analogues were glutamic acid, proline, leucine, and aspartic acid, according to Chiang’s study [22], and the amount of cysteine in the meat replacements was higher than that of firm tofu and steamed chicken. It has also been discovered that adding green tea to TVP improved texturization and antioxidant properties but had a negative effect on the expansion and NSI [90]. Recently, Sakai et al. [26] and Chen et al. [21] investigated the in vitro gastrointestinal digestibility of meat analogues. It was reported that HME could improve protein digestibility in several protein materials [21]. It seems that the extrusion field in this phase focused on finding approaches to mimic the structure of the meat. The next phase will also consider the amino acid profile or protein quality (PDCAAS), but we are not there yet. This follows a logical order, since there is no need to optimize the nutrition profile if the materials do not fulfill the texture criteria.

##### Cooking Quality

Cooking quality in this review was related to analyses performed before and after extrusion, such as cooking loss or frying loss, swelling index, water absorption capacity (WAC), and breakage rate. The percentage difference between the weight of the sample before and after cooking is referred to as “cooking loss”, and it is an essential indicator in determining the quality of the meat analogues in relation to the amount of juice it retains and the amount of product it produces overall [26]. In general, preparation factors such as composite materials affect cooking loss in processed meat products [50]. For instance, in mushroom sausage, the addition of all types of binding agents decreased cooking losses, with carrageenan giving the best results, followed by xanthan gum, soy protein concentrates, and casein [66]. According to Neumann [95], the water absorption capacity of a product correlated with its texture after rehydration. Lin and colleagues [62] studied water absorption capacity in the extruded meat analogues and discovered that samples extruded at the high moisture content (70%) and high cooking temperatures (149 °C and 160 °C) had the highest WAC. Furthermore, the authors reported that the porosity of meat analogues influenced the WAC, since extrudates with comparable physical structures and similar moisture contents did not significantly vary in WAC.

##### Sensory Evaluation

Another important test that many researchers employed to confirm that the products are acceptable from the consumer’s perspective is the sensory evaluation. The sensory qualities of the generated meat analogues can be evaluated using descriptive sensory analysis and the hedonic scale. A scale or a test using hedonic 7-point, 9-point, and 11-point scales was used to achieve this by untrained, semi-trained, or trained panelists. De Angelis et al. [49] used an 11-point structured scale ranging from 0 to 10, and the sensory assessment emphasized a powerful odor and taste profile of dry-fractionated pea protein and oat protein (PDF–OP), whereas the extrudates generated by protein isolates had neutral sensory features. It was found in this review that sensory assessment is not included in many investigations of meat analogues; most of them are studies on restructured meat substitutes. Sensory evaluation was conducted by Grahl et al. [76] using conventional profiling on spirulina and soy-based meat analogues to measure the intensities and amplitudes of the feelings as well as to subjectively characterize the samples. Rousta et al. [56] evaluated the texture of patties manufactured from *Aspergillus oryzae* biomass (edible fungi) and compared them with two other commercial patties in Sweden, namely, Beyond and Quorn. The study also revealed that restructured meat could give a different taste because of the chemicals and enzymes used in the pre-treatments, which degrade carbohydrates, proteins, and fats into parts [56].

##### Other Assessments

In this sub-theme, several assessments were found to be used in the reviewed studies, such as volatile compounds [52], microbiological evaluation of meat analogues [75], and life cycle assessment of products produced from meat analogues [104]. Kaleda et al. [52] found that extrusion decreased volatile compounds due to high temperature (150 °C). The microbiological evaluation of meat analogues products was discovered to be a crucial assessment to regulate the microbial growth of the meat analogues and at all stages of processing as well as to estimate the product’s shelf life. Filho et al. [75] analyzed the properties of raw materials, evaluated the microbial limit testing of the canned product before retorting it, and investigated the most critical processing stages to exert control over the growth of microorganisms. However, to go on to the next phase, researchers must first complete the assessments described above, and that will provide a good understanding of each material and the fundamental processing approach.

## 4. Recommendation for Future Studies

The findings of this review made it evident that a significant amount of research has been conducted on developing better meat substitutes by using improved texturization methods. The recent trend in publishing indicates that technology in developing meat analogues is receiving greater attention. Even though methods involving extrusion are being employed on an industrial scale, these processes are still in their formative stages and have great room for improvement. Fiber and protein–protein interaction research were less prevalent than in other subjects. However, several attempts were found, such as morphology development (cryo-imaging) and flow characteristics (closed-cavity rheometer) with online sensors and simulations showing a promising future in this area. For high-moisture extrusion technology to be used efficiently, researchers need to have a solid understanding of how changes in the conformation of macromolecules occur and an understanding of the mechanism behind it. This is due to a limited number of studies that have been conducted to evaluate and understand the texturization methods that are now in use. Regarding textural features, researchers should consider what texture they should aim for, including the cooking methods. It is also necessary to conduct more studies on physical structuring approaches that have shown potential, such as extensional shearing devices and high-pressure processing, to diversify methods or improve existing methods.

With the passage of time and the growth of knowledge, several new start-up businesses such as Beyond Meat, Meatless, and Impossible Foods, as well as some popular brands such as Ikea, are concentrating on capturing the meat analogues market worldwide. In this review, sensory assessment and nutritional research on meat analogues were less common than in other sub-themes. Sensory experiments on novel plant-based protein meat analogues might be carried out to determine the market acceptance of each product. Despite the variety of novel plant proteins, additional knowledge is required on the protein powder components that govern extrudability. Future studies on the absorption rate in the gastrointestinal system should also be conducted to ensure that the human body can absorb the nutrients in the produced meat analogues.

## 5. Conclusions

In this review, we conducted a systematic review and bibliometric analysis of the literature on 84 articles published between 1972 and January 2022. Two research questions were addressed: (RQ1) To what extent has research been conducted on the development of meat analogues derived from plant protein-based materials? The response to this question was presented as a set of findings in Section 3.1, Section 3.2 and Section 3.3 in descriptive analysis; (RQ2) What are the key research themes in the literature on plant-based meat analogues? The answer to this question was given in the discussion part of Section 3.4, where we divided our findings into six different themes: (i) objectives of the study; (ii) type of plant protein; (iii) product type; (iii) added ingredients; (iv) texturization technique; and (v) quality assessment.

While this review study has been conducted comprehensively, there are several practical limitations, as mentioned in other studies. Despite the fact that Scopus and WoS are two of the most widely used databases, there are still many journals that have not been indexed. Even though we employed a broad search string, it is possible that some studies were missed by our review. There is a chance that not all journals were included in the search, since no search term is 100% accurate. In future literature reviews, using additional search engines or databases may benefit the broad overview. In addition, this study did not include languages other than English, which might be seen as a source of bias, particularly for publications conducted in non-western cultures.

This study shows how research on meat analogues shifted from focusing on primary components, principally soy, to novel protein alternatives, complete and partial, and lastly to more advanced materials. It was apparent how research moved from individual relationships between protein to multidimensional and integrative research on protein and its chemical changes and structures, and protein–protein interactions during thermal–mechanical processing. This advancement permitted the inclusion of a more extensive range of issues based on plant protein beyond animal flesh. Future studies should focus more on fibers, protein–protein interactions, and macromolecule conformations and mechanisms, diversifying or improving current methods, sensory attributes, and the gastrointestinal absorption rate of each novel protein ingredient.

## Figures and Tables

**Table 1:** Search string used in the selected database.

Database	Search String
**SCOPUS**	TITLE-ABS-KEY (((“meat analog*” OR “meat substitute*” OR “meat replace*” OR “meat alternat*” OR “mock meat” OR “imitat* meat” OR “faux meat” OR “HMMA” OR “TVP” OR “vegan meat” OR “plant based meat” OR “textur* protein” OR “extrud* protein” OR “extrud* food”) AND (“extru*” OR “textur*”)) AND NOT (“drug” OR “polymer*” OR “packaging” OR “DNA” OR “gene*” OR “pet food” OR “animal feed”)) AND (LIMIT-TO(LANGUAGE, “English”)) AND (LIMIT-TO (SRCTYPE, “j”))
**Web of Science** **(WoS)**	TS=(((“meat analog*” OR “meat substitute*” OR “meat replace*” OR “meat alternat*” OR “mock meat” OR “imitat* meat” OR “faux meat” OR “HMMA” OR “TVP” OR “vegan meat” OR “plant based meat” OR “textur* protein” OR “extrud* protein” OR “extrud* food”) AND (“extru*” OR “textur*”)) NOT (“drug” OR “polymer*” OR “packaging” OR “DNA” OR “gene*” OR “pet food” OR “animal feed”)) AND (LIMIT-TO(LANGUAGE, “English”)) AND (LIMIT-TO(SRCTYPE, “j”))

* Truncation of terms was used to capture variation in language.

**Table 2:** Journals with the largest number of documents.

Journal Name	Number of Publications	Quartile and Indexed by	
		Scopus	WoS
*Foods*	15	Q1	Q1
*Journal of Food Science*	9	Q2	Q2
*Journal of the Science of Food and Agriculture*	6	Q1	Q1
*Journal of Food Engineering*	8	Q1	Q1
*LWT-Food Science and Technology*	4	Q1	Q1
*Food Science and Biotechnology*	3	Q2	Q3
*Innovative Food Science and Emerging Technologies*	3	Q1	Q1
*Food Research*	2	Q3	Q3
*Food Structure*	2	Q1	Q2
*International Journal of Food Science and Technology*	2	Q1	Q2
*Journal of Agricultural and Food Chemistry*	2	Q1	Q1
*Food Hydrocolloid*	2	Q1	Q1
*Journal of Cleaner Production*	2	Q1	Q1

**Table 3:** Findings from 84 reviewed articles.

Authors (Year)/Theme	Objectives of Study				Type of Plant Protein		Product Type		Added Ingredient			Texturization Technique					Quality Assessment						
Sub-themes	TT	M	FF	OP	SP	OPP	TVP	MA	BA	F	OI	SSE	TSE	SC	S	M/O	C/FP	PP	FF	NA	CQ	SE	OA
Aguilera et al. (1980) [20]		√		√		√	√	√				√					√	√	√		√	√	
Arora et al. (2017) [66]		√				√		√	√		√					√	√	√		√	√	√	
Arueya et al. (2017) [67]	√	√				√	√					√					√	√		√		√	
Bakhsh et al. (2021a) [41]				√	√	√		√	√	√	√					√	√	√			√	√	
Bakhsh et al. (2021b) [68]				√		√		√	√	√	√					√	√	√			√	√	
Bayram et al. (2007) [69]		√				√		√		√	√					√	√	√				√	
Bruckner et al. (1987) [70]	√	√			√	√	√	√				√					√				√		
Byun et al. (1978) [12]	√		√		√		√								√			√					
Caporgno et al. (2020) [71]	√	√			√	√		√					√				√	√	√	√			
Chen et al. (2021) [21]	√	√	√			√		√			√		√					√	√	√			
Chiang et al. (2019) [72]		√		√	√	√		√			√		√				√	√	√	√		√	
Chiang et al. (2021) [22]	√			√	√	√		√			√					√	√	√	√	√			
Cornet et al. (2021) [23]				√	√	√		√			√			√			√						
Dahl and Villota (1991) [60]		√			√			√					√				√		√				
De Angelis et al. (2020) [49]	√	√		√	√	√		√					√				√	√				√	
Emin et al. (2017) [73]				√		√		√								CCR	√						
Fang et al. (2014) [74]	√			√	√			√					√				√	√					
Ferawati et al. (2021) [24]		√				√		√					√				√	√					
Filho et al. (2005) [75]		√		√	√	√	√	√		√	√					√							√
Gihyun et al. (2020) [50]		√			√	√		√			√					√	√	√	√		√	√	
Grahl et al. (2018) [76]	√	√			√	√		√					√					√				√	
Hashizume (1978) [19]	√				√		√									FR	√						
Husain and Huda-F. (2020) [77]		√				√		√	√							√	√	√			√		
Immonen et al. (2021) [51]	√		√			√		√			√		√				√	√	√				
Jia et al. (2021) [78]		√	√			√		√						√			√	√	√				
Kaleda et al. (2020) [52]		√		√		√		√					√				√	√		√		√	
Kamani et al. (2019) [79]		√			√	√		√	√	√	√					√	√	√			√	√	
Kendler et al. (2021) [53]				√		√		√		√			√					√	√				
Kim et al. (2011) [80]		√				√		√	√							√	√	√	√	√			
Kozlowska et al. (1979) [81]	√			√	√	√	√					√							√				
Krintiras et al. (2014) [82]	√		√		√	√		√						√				√	√				
Krintiras et al. (2015) [83]	√				√	√		√						√				√	√				
Krintiras et al. (2016) [17]	√		√		√	√		√						√				√	√				
Lee et al. (2005) [84]	√			√	√			√					√										√
Lee et al. (2022) [85]		√		√	√	√		√					√				√	√		√			
Lee and Hong (2020) [86]					√	√		√	√								√	√	√		√		
Lin et al. (2000) [61]				√	√			√					√				√	√					
Lin et al. (2002) [62]	√			√	√			√									√		√			√	
Lindriati et al. (2020) [87]					√	√		√								√	√	√					
Liu and Hsieh (2007) [88]				√	√	√		√					√				√	√					
Liu and Hsieh (2008) [89]				√	√	√		√					√				√						
Liu et al. (2021) [54]	√	√				√		√					√				√	√					
Ma and Ryu (2019) [90]		√			√	√	√				√		√				√	√	√				
Mattice and Marangoni (2020) [91]	√					√		√			√				√	AS/ME	√	√	√				
Maung et al. (2020) [92]		√			√	√	√				√		√			√	√	√	√				
Mazlan et al. (2020) [55]	√	√			√	√		√		√	√	√					√	√	√				
Murillo et al. (2019) [93]	√	√	√			√		√					√					√	√				
Nayak and Panda (2016) [94]		√				√		√								√	√	√					
Neumann et al. (1984) [95]	√	√			√	√	√	√				√					√		√				
Omohimi et al. (2014) [96]	√	√				√		√				√					√	√					
Osen et al. (2015) [32]	√	√		√		√		√					√				√						
Osen et al. (2014) [31]	√	√				√		√					√					√	√				
Palanisamy et al. (2019) [97]	√	√				√		√					√				√	√	√		√		
Palanisamy et al. (2018) [98]	√	√	√	√	√											PRE	√	√	√		√	√	
Parmer and Wang (2004) [99]	√	√		√	√	√		√				√					√	√					
Pietsch et al. (2019) [100]	√			√	√			√					√				√	√	√				
Pori et al. (2022) [101]				√		√		√			√		√				√	√					
R. Alonso et al. (2000) [102]		√		√		√		√					√				√						
Ranasinghesagara et al. (2006) [64]	√	√	√		√	√		√					√						√				
Ranasinghesagara et al. (2009) [25]	√	√	√		√	√		√					√						√				
Ranasinghesagara et al. (2005) [63]	√	√	√		√	√		√					√						√				
Rehrah et al. (2009) [103]	√	√				√		√				√					√	√				√	
Rousta et al. (2021) [56]		√				√		√			√					√	√					√	
Saerens et al. (2021) [104]	√	√		√	√	√	√	√	√	√	√		√			√							√
Sakai et al. (2021) [26]		√		√	√	√		√	√	√	√					√	√	√		√	√		
Saldanha do Carmo et al. (2021) [105]		√				√		√					√				√	√				√	
Samard et al. (2019) [106]	√	√			√	√		√					√				√	√					
Samard and Ryu (2019) [27]	√	√			√	√	√						√				√	√	√	√			
Sharima et al. (2018) [107]		√		√		√		√								√	√	√		√	√	√	
Stanley et al. (1972) [108]																							
Stephan et al. (2018) [109]		√				√		√			√					√	√	√				√	
Taranto et al. (1978) [11]	√					√		√					√			√		√	√				
Wen et al. (2017) [110]		√	√			√	√				√		√				√	√	√				
Wittek et al. (2021a) [58]			√		√			√					√				√	√	√				
Wittek et al. (2021b) [59]			√		√			√					√					√	√				
Wu et al. (2018) [111]	√				√	√	√						√				√	√	√				
Xia et al. (2022) [112]		√				√		√					√				√	√	√				
Yao et al. (2004) [65]			√		√	√		√					√					√	√				
Yuan et al. (2022) [57]		√			√	√	√	√	√	√	√	√				√	√	√	√			√	
Yuliarti et al. (2021) [113]						√		√			√					FR	√	√	√			√	
Zahari et al. (2020) [15]	√	√			√	√		√					√				√	√					
Zahari et al. (2021) [14]		√				√		√					√				√	√		√			
Zhang et al. (2019) [114]		√	√			√		√					√					√	√				
Zhang et al. (2020) [115]		√	√	√		√		√			√		√				√	√	√				
**Objectives of Study**	**Type of Plant Protein**						**Product Type**					**Added Ingredient**			**Texturization Technique**					**Quality Assessment**			
TT = texturization technique M = materials/ingredients FF = fiber formation OP = other properties (functional/protein interaction)	SP = soy protein OPP = other plant proteins (oilseed, mushroom, legumes)						TVP = texturized vegetable protein MA = meat analogues					BA = binding agent F = fat OI = other ingredients			SSE = single screw extrusion TSE = twin screw extrusion SC = shear/Couette cell S = spinning M/O = mixing/other methods (CCR = closed-cavity rheometer) (F = fermentation) (AS = antisolvent precipitation) (ME = mechanical elongation) (FR = freezing) (PRE = planetary roller extruder)					C/FP = chemical/functional properties PP = physical properties FF = fiber formation NA = nutritional analysis CQ = cooking quality SE = sensory evaluation OA = other assessment			
